# Rhizobia mitigates autotoxicity in *Malus hupehensis* via phenolic degradation and enhanced melatonin-antioxidant responses

**DOI:** 10.3389/fmicb.2026.1912880

**Published:** 2026-08-13

**Authors:** Cun-cui Kong, Miao Zhang, Min-min Xu, Hong-Xia Zhang, Song Qin, Cheng-gang Ren

**Affiliations:** 1 College of Horticulture, Ludong University, Yantai, Shandong, China; 2 Comprehensive Technical Service Center of Zibo Customs, Zibo, China; 3 Shandong Provincial Academy of Environmental Sciences Co., Ltd., Jinan, China; 4 Key Laboratory of Coastal Zone Biology and Biological Resources Utilization, Yantai Institute of Coastal Zone, Chinese Academy of Sciences, Yantai, Shandong, China

**Keywords:** autotoxicity, melatonin, phenolic-degrading rhizobia, phloridzin, rhizosphere microbiome, ROS

## Abstract

**Introduction:**

Phloridzin is a major autotoxin contributing to apple replant disease (ARD) by inducing oxidative stress and disrupting the rhizosphere ecology.

**Methods:**

This study evaluated the potential of *Ensifer meliloti* y5077 to mitigate these detrimental effects in *Malus hupehensis*.

**Results:**

Strain y5077 demonstrated high degradation efficiency, removing 100% of phloridzin *in vitro* within 48 h and reducing soil phloridzin levels by 68.4% in 60-day pot experiments. Inoculation significantly improved seedling growth under autotoxic stress, with root dry weight increased compared to non-inoculated stressed plants. These growth benefits were linked to a significant increase in endogenous melatonin levels and the restoration of antioxidant enzyme activities to normal basal levels, which effectively suppressed malondialdehyde (MDA) and reactive oxygen species (ROS) accumulation. Furthermore, y5077 inoculation restructured the rhizosphere microbiome, characterized by significant shifts in the relative abundance of specific microbial taxa (e.g., increases in *Firmicutes* and *Actinobacteriota*, and decreases in *Basidiomycota*).

**Discussion:**

These results demonstrate that y5077 acts as a multi-functional bioremediator that integrates toxin degradation with physiological priming and rhizosphere microbial community restructuring, providing a robust and sustainable biological solution for mitigating phloridzin-induced autotoxic stress in apple seedlings.

## 1 Background

China plays a pivotal role in the global apple industry, being the largest producer and exporter of fresh apples. However, sustainable apple production is severely constrained by the widespread occurrence of Apple Replant Disease (ARD), which arises due to limited arable land and difficulties in crop rotation. ARD manifests as stunted growth, reduced biomass, and yield decline in apple trees repeatedly cultivated on the same site. Its etiology is complex, involving deteriorations in soil chemical properties (e.g., accumulation of autotoxic compounds) and microbial community imbalances (such as reduced beneficial microbes and increased pathogens) (Somera and Mazzola, 2022).

Research indicates that phenolic compounds released during the decomposition of apple tree residues (e.g., leaf litter and roots) are major contributors to ARD (Duan et al., 2025; Jiang et al., 2022; Radl et al., 2019). Among these, phloridzin—a dihydrochalcone glycoside abundant in apples—has been demonstrated to significantly inhibit the growth of apple seedling (Du et al., 2024; Zhou et al., 2019). High concentrations of phloridzin not only reduce photosynthetic and transpiration rates but also disrupt energy metabolism and carbon assimilation by inhibiting key enzymes in chloroplast photophosphorylation and the tricarboxylic acid (TCA) cycle, such as citrate synthase and malate dehydrogenase (Xiang et al., 2021). According to Wang et al., soils in severely ARD-affected orchards can contain phloridzin at concentrations up to approximately 6 mg·kg^−1^ (Wang et al., 2015). The toxicity mechanism is closely associated with a burst of reactive oxygen species (ROS). Oxidative stress induced by phenolic compounds not only directly damages macromolecules like proteins and nucleic acids but also causes secondary damage through lipid peroxidation products such as malondialdehyde (MDA) (Sharifi-Rad et al., 2020; Zhang et al., 2021). Therefore, targeting the persistent autotoxin phloridzin for microbial decomposition presents a logical and promising approach to interrupt the cycle of soil sickness and mitigate ARD, paving the way for the application of functional microorganisms.

Rhizobia are a type of plant growth-promoting rhizobacteria commonly utilized as biofertilizers. Notably, they can establish mutually beneficial relationships with non-leguminous plants without inducing root nodule formation (Pankievicz et al., 2019). This interaction highlights the multifaceted role of rhizobia in enhancing plant health and productivity beyond leguminous species. Studies report that numerous non-leguminous plants, such as rice, maize, and spinach, can benefit from rhizobia as growth-promoting bacteria (Bloch et al., 2020; Wu et al., 2018). Rhizobia enhance the growth of non-leguminous plants through direct mechanisms, including the stimulation of phytohormone production and improved availability of insoluble phosphorus (Wasai-Hara et al., 2020). Additionally, by boosting the activity of key antioxidant enzymes—superoxide dismutase (SOD), peroxidase (POX), and catalase (CAT)—rhizobia help mitigate oxidative damage caused by reactive oxygen species (ROS) generated under biotic and abiotic stresses (Tokumoto et al., 2020). Furthermore, it is widely recognized that rhizobia play a significant role in degrading aromatic pollutants, such as phenolic compounds (Damaj and Ahmad, 1996; Golubev et al., 2024; Ren et al., 2017).

Recently, melatonin has been recognized as a versatile biological molecule with the potential to alleviate both abiotic and biotic stresses in plants (Huang and Jin, 2024; Khalofah, 2024; Wang et al., 2025). Accumulating evidence indicates that melatonin effectively neutralizes ROS, protecting plants from oxidative damage (Pardo-Hernández et al., 2020). Research suggests that melatonin may also help maintain oxidative homeostasis under abiotic stress by modulating photorespiration and regulating carbon and nitrogen metabolism (Efimova et al., 2023; Qari et al., 2022). Interestingly, a recent study found that melatonin can comprehensively alleviate apple replant disease by reducing phloridzin levels in the rhizosphere soil and roots, thereby improving the rhizosphere microenvironment and shaping the endophytic microbial community structure (Ma et al., 2024). Related studies also report that certain non-rhizobial endophytes can stimulate melatonin production in grapevines, effectively reducing ROS-induced damage (Jiao et al., 2016). However, the interactive network and synergistic regulatory mechanisms involving melatonin, phloridzin, and rhizobia remain to be fully elucidated.

Recent advances have highlighted the potential of specific rhizobacteria in mitigating replant obstacles. However, current research progress predominantly focuses on evaluating these strains either purely as passive degraders of phenolic autotoxins or as general plant growth promoters in isolation. A significant knowledge gap remains regarding the holistic interplay between targeted microbial degradation, the regulation of plant endogenous signaling networks (such as melatonin pathways), and the subsequent restructuring of the rhizosphere microbiome under severe autotoxic stress.

To bridge this gap, the novelty of this study lies in transitioning the paradigm from single-function remediation to a multi-trophic, integrated systemic restoration. We hypothesized that *Ensifer meliloti* y5077 functions beyond a simple toxin degrader, acting as a dual-action bioremediator. Therefore, the primary goals of this research were: (1) to evaluate the high-efficiency degradation of phloridzin by y5077 and its resulting alleviation of oxidative stress in *Malus hupehensis*; (2) to uncover the novel mechanistic link between rhizobial colonization and the induction of melatonin associated antioxidant defenses; and (3) to elucidate how this plant-microbe synergy drives the ecological rebalancing of the rhizosphere microbiome.

## 2 Results

### 2.1 Strain selection and colonization

In this experiment, four rhizobia strains, *Ensifer Meliloti* y4071, y5077, and *Ensifer arboris* y4009, y4031, with the capacity to degrade 10 μM phloridzin within 96 h (degradation rate >80%), were subjected to higher concentrations of phloridzin in shake flasks. The results showed the ability of *E. arboris* y4009 and *E. arboris* y4031 to degrade phloridzin was limited at higher concentrations (> 20 μM). The degradation efficiency of *E. meliloti* y4071 can reach over 50%. Meantime, *E. meliloti* y5077 had the highest degradation rate, up to 100% (Supplementary Figure S1). Furthermore, tests were carried out in autoclaved soil to evaluate the degradation efficiency of *E. meliloti* y5077 on phloridzin in planting conditions in subsequent experiments. The results showed that in the soil containing 50 μmol kg^−1^ phloridzin, the amount of residual phloridzin was less than 0.2 μmol kg^−1^ in the *E. meliloti* y5077 treated group, while the residual amount of phloridzin in the planting group was 5.4–14.5 μmol kg^−1^ (Supplementary Table S1).

To evaluate the vitality of rhizobia in the presence of phloridzin in the soils, root colonization rates were recorded. As shown in Supplementary Table S2, the root colonization rates of four rhizobia were close without phloridzin treatment. But it was higher for *E. meliloti* y5077 compared to the other strains under phloridzin treatment. The data implied that a high concentration of phloridzin might also have toxic effects on rhizobia, which limited the bacterial colonization on *M. hupehensis* root. And *E. meliloti* y5077 had the highest tolerance to phloridzin toxicity in this study. Taken together, the foregoing results suggested the ability of *Ensifer* strains to withstand the toxicity of phenolic acids and to utilize phenolic acids varied widely among the strains.

**Figure 1:**
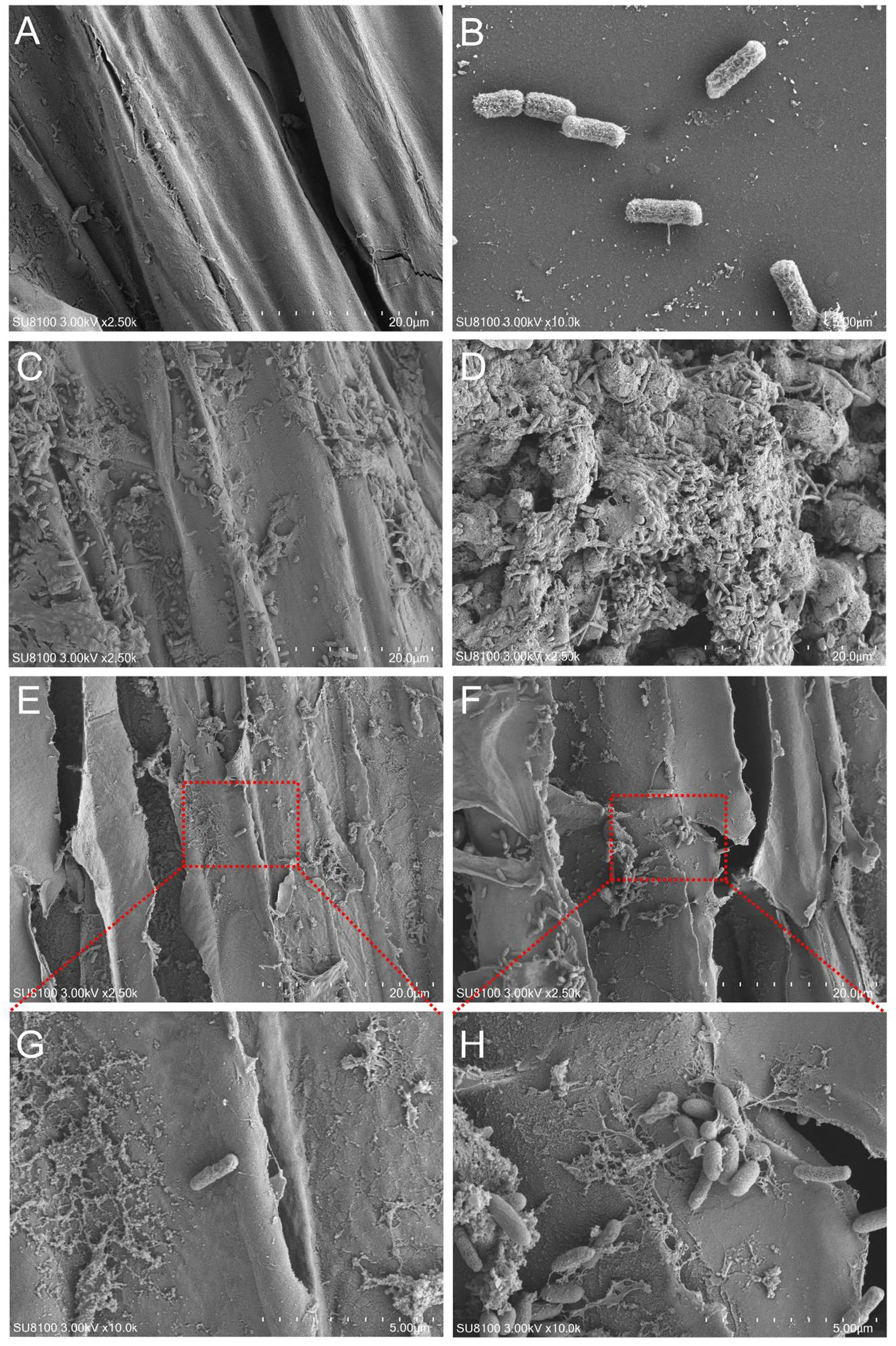
SEM analysis of *Ensifer meliloti* y5077 and its colonization on *M. hupehensis* root. **(A)** SEM image of uninoculated root surfaces, appearing clean and devoid of visible bacterial cells. **(B)** SEM image of the *E. meliloti* y5077 pure culture illustrating its characteristic short-rod morphology. **(C,D)** Obvious colonization of y5077 on the root surface without phloridzin **(C)**, and the formation of dense bacterial clusters and biofilm-like structures under 100 μmol/kg phloridzin stress **(D)**. **(E,F)** Attachment patterns of rhizobial cells observed on the longitudinal sections of the roots under treatments without phloridzin **(E)** and with phloridzin **(F)** (magnification: 2.50 k; scale bar = 20.0 μm). **(G,H)** Detailed view of the typical rod-shaped morphology of strain y5077 and its attachment details on the longitudinal root sections under treatments without phloridzin **(G)** and with phloridzin **(H)** (magnification: 10.0 k; scale bar = 5.00 μm). All micrographs were captured using a Hitachi SU8100 SEM at an accelerating voltage of 3.00 kV.

Strain y5077 exhibited the highest degradation efficiency among the tested isolates. To further verify its colonization capacity, we evaluated the distribution of y5077 in the rhizosphere and on the root surfaces of *M. hupehensis* under both control and phloridzin-stress conditions. As shown in Supplementary Table S2, the colonization density of y5077 in the root surface was approximately 1.62 × 10^5^ under control conditions. Notably, treatment with 100 μmol kg^−1^ phloridzin did not inhibit y5077; instead, the strain demonstrated a superior competitive advantage, with its colonization on the root surface significantly increasing to 2.82 × 10^7^. Scanning electron microscopy (SEM) further confirmed these results: while y5077 was sparsely scattered on the root surface without phloridzin (Figure 1A), it formed dense bacterial clusters and biofilm-like structures firmly adhering to the root surface and longitudinal sections under phloridzin stress (Figures 1B,D,F). This robust colonization capacity serves as the biological foundation for y5077 to sustain its degradative activity and exert plant growth-promoting functions under adverse conditions.

### 2.2 The protective effect of rhizobia on *M. hupehensis* under phloridzin stress

**Figure 2:**
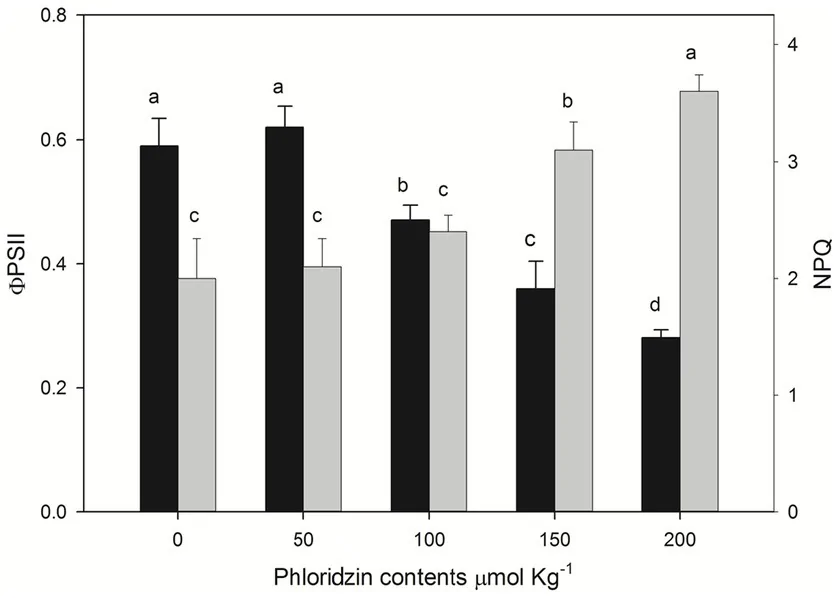
Effect of different phloridzin concentrations on photosynthetic parameters of *M. hupehensis*. Left, photosystem II efficiency (ΦPSII); right, non-photochemical quenching of chlorophyll fluorescence (NPQ). Values are means of three independent experiments. The error bar represents the standard error. Data are the means of three replicates (SD). Different letters above the error bars indicate statistical significance at *p* < 0.05.

The autotoxicity induced by phloridzin significantly inhibited the growth and development of *M. hupehensis* seedlings. Treatment with 100 μmol kg^−1^ phloridzin led to a marked reduction in plant height, ground diameter, and biomass compared to the control. Specifically, the total biomass in the phloridzin group decreased by 11.86% relative to the control. Furthermore, phloridzin stress severely impaired root architecture, resulting in significantly shorter primary roots and reduced lateral root density (Tables 1, Supplementary Table S3, Supplementary Figure S2). On the other hand, phloridzin also affected the photosynthetic capacity of *M. hupehensis* plants (Figure 2). The inhibitory effect began to show in 100 μmol kg^−1^ phloridzin-treated soil; the degree of inhibition increases with increasing concentration of phloridzin in soil.

**Table 1:** Biomass (g dry weight pot^−1^) and root shoot ratio (Wroot/Wshoot) of different concentrations phloridzin soils.

Phloridzin content in soil μmol kg^−1^						
		0	50	100	150	200
Control	Shoot	9.41 ± 1.02d	9.66 ± 0.23d	8.16 ± 0.23c	6.25 ± 0.36b	4.63 ± 0.4a
	Root	12.35 ± 0.58c	12.42 ± 0.13c	11.02 ± 0.33b	10.38 ± 0.25b	7.56 ± 0.73a
	W_root_/W_shoot_	0.76	0.67	0.67	0.60	0.53
y5077	Shoot	9.67 ± 0.52b	9.31 ± 0.35b	9.7 ± 0.81b	8.94 ± 1.2b	7.41 ± 1.02a
	Root	14.34 ± 0.7b	12.72 ± 0.26b	13.54 ± 0.92b	12.36 ± 1.58b	10.35 ± 0.48a
	W_root_/W_shoot_	0.67	0.73	0.71	0.72	0.71

Means with different letters within each row are significantly different (one-way ANOVA, *p* < 0.05).

However, inoculation with *E. meliloti* y5077 effectively mitigated these inhibitory effects. In the 100 μmol kg^−1^ phloridzin group, seedling growth parameters were significantly improved compared to the control group, with total biomass increasing by 11.77%, respectively, nearly reaching the levels observed in the control (Table 1). This growth recovery was closely associated with the restoration of root development, as y5077-treated seedlings maintained robust root systems even under phloridzin stress.

**Figure 3:**
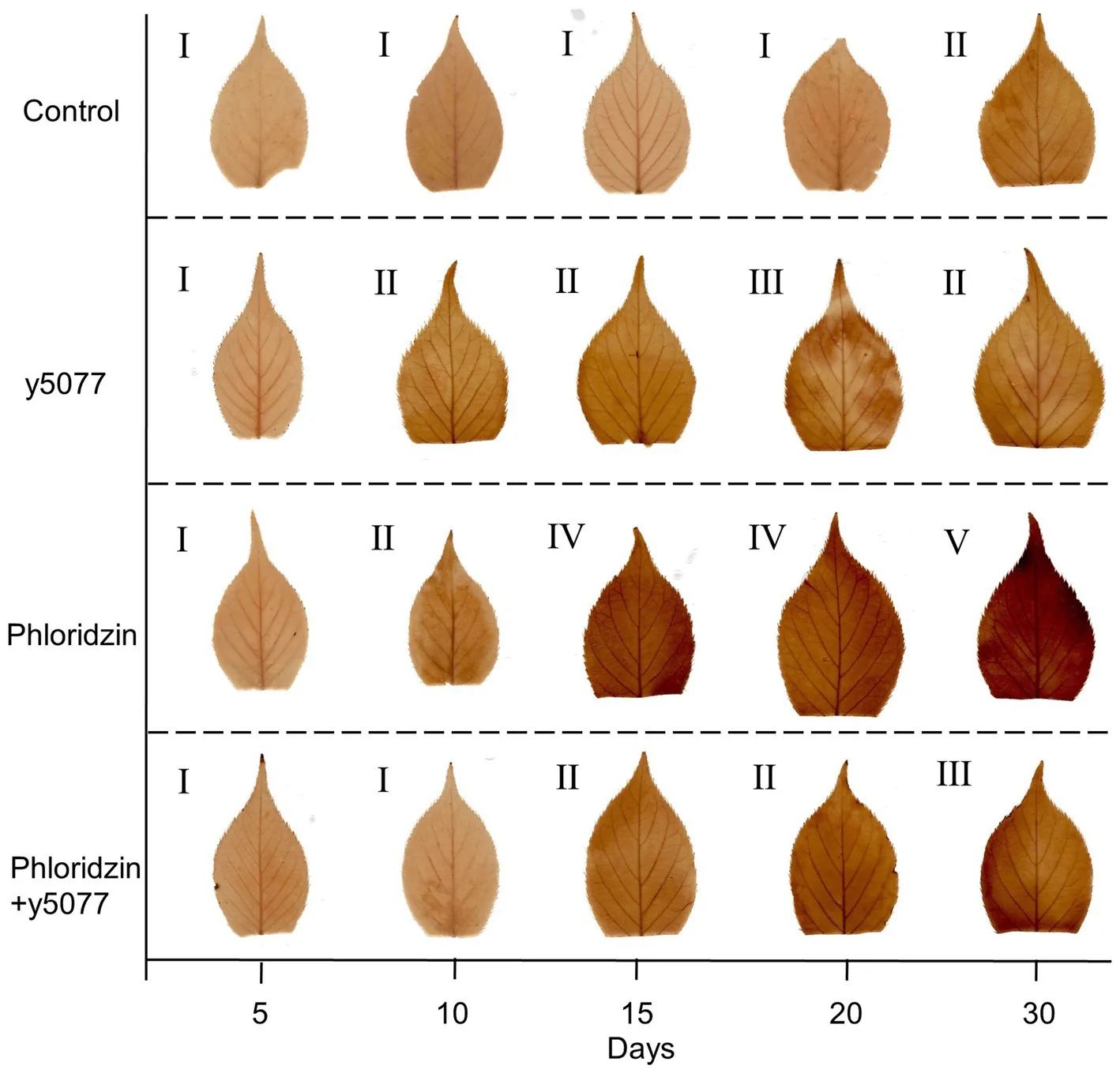
*In situ* detection of H_2_O_2_ accumulation in *M. hupehensis* leaves via DAB staining. The accumulation of H_2_O_2_, indicated by reddish-brown precipitates, was used to evaluate the oxidative stress levels in seedlings over a 30-day period. Roman numerals (I–V) represent the qualitative staining intensity levels: Level I (no pigment deposition), Level II (slight brown deposition), Level III (heavy brown deposition), Level IV (entire leaf stained brown), and Level V (localized intense brown-black staining). Qualitative scoring was performed blindly. Quantitative validation of the stained areas was performed using ImageJ, provided in Supplementary Table S7.

**Figure 4:**
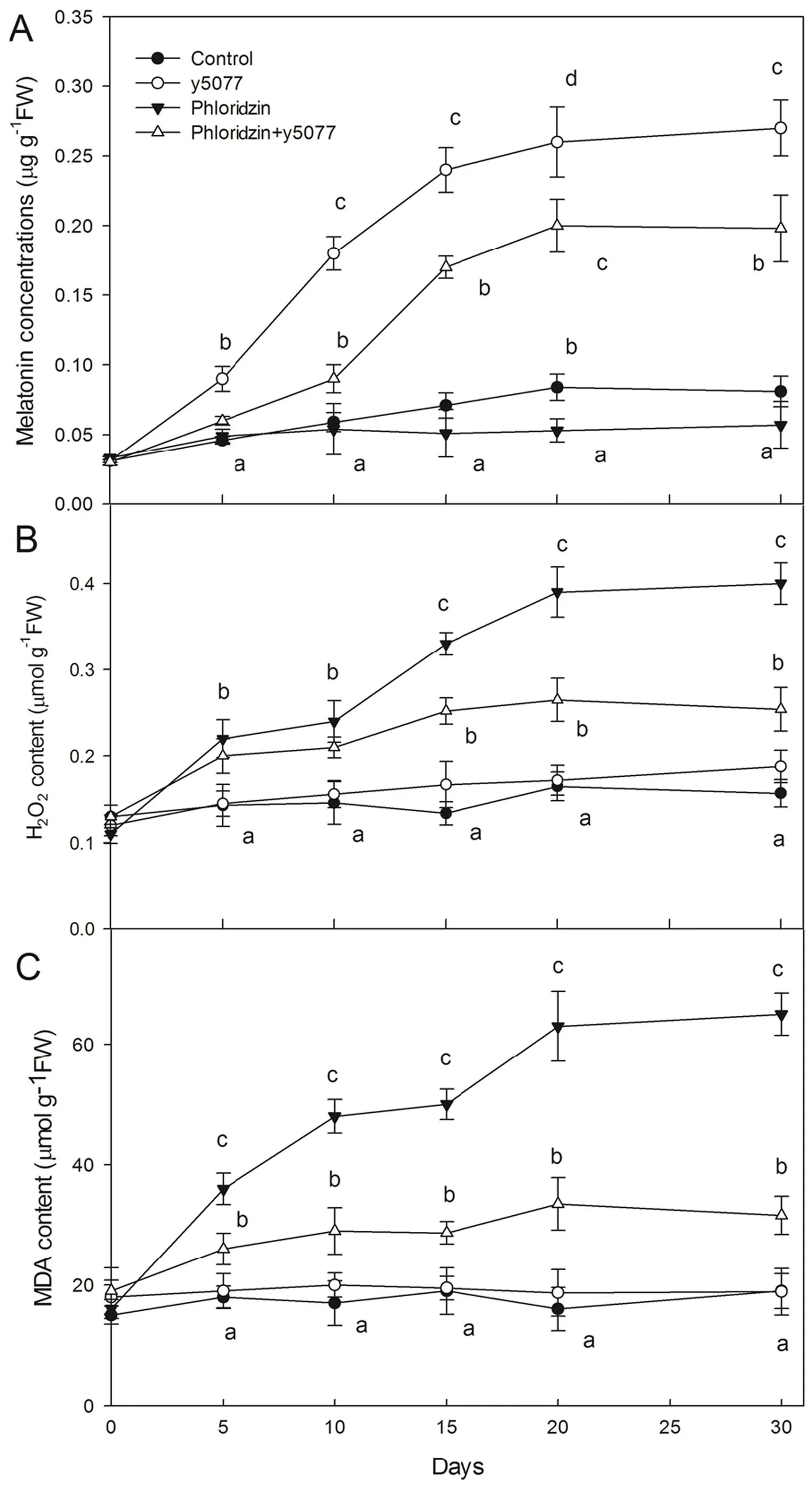
Effect of rhizobia inoculation on melatonin, H_2_O_2_, and MDA content in *M. hupehensis* under phloridzin stress. **(A)** Contents of melatonin; **(B)** contents of H_2_O_2_; **(C)** contents of MDA. Phloridzin concentration applied in the experiment was 100 μmol kg^−1^). Data were analyzed using Two-way ANOVA followed by Tukey’s HSD test (*n* = 3). Statistical evaluation by Two-way ANOVA indicates significant main effects and interaction terms for all parameters: Melatonin (Treatment: *F*_3,24_ = 142.58, *p* < 0.001; Time: *F*_3,24_ = 89.34, *p* < 0.001; Treatment × Time: *F*_9,24_ = 28.65, *p* < 0.001); H_2_O_2_ (Treatment: *F*_3,24_ = 95.16, *p* < 0.001; Time: *F*_3,24_ = 51.20, *p* < 0.001; Treatment × Time: *F*_9,24_ = 14.32, *p* < 0.001); MDA (Treatment: *F*_3,24_ = 118.42, *p* < 0.001; Time: *F*_3,24_ = 64.12, *p* < 0.001; Treatment × Time: *F*_9,24_ = 19.78, *p* < 0.001). Different lowercase letters indicate significant differences between treatments at *p* < 0.05 (Tukey’s HSD test).

**Figure 5:**
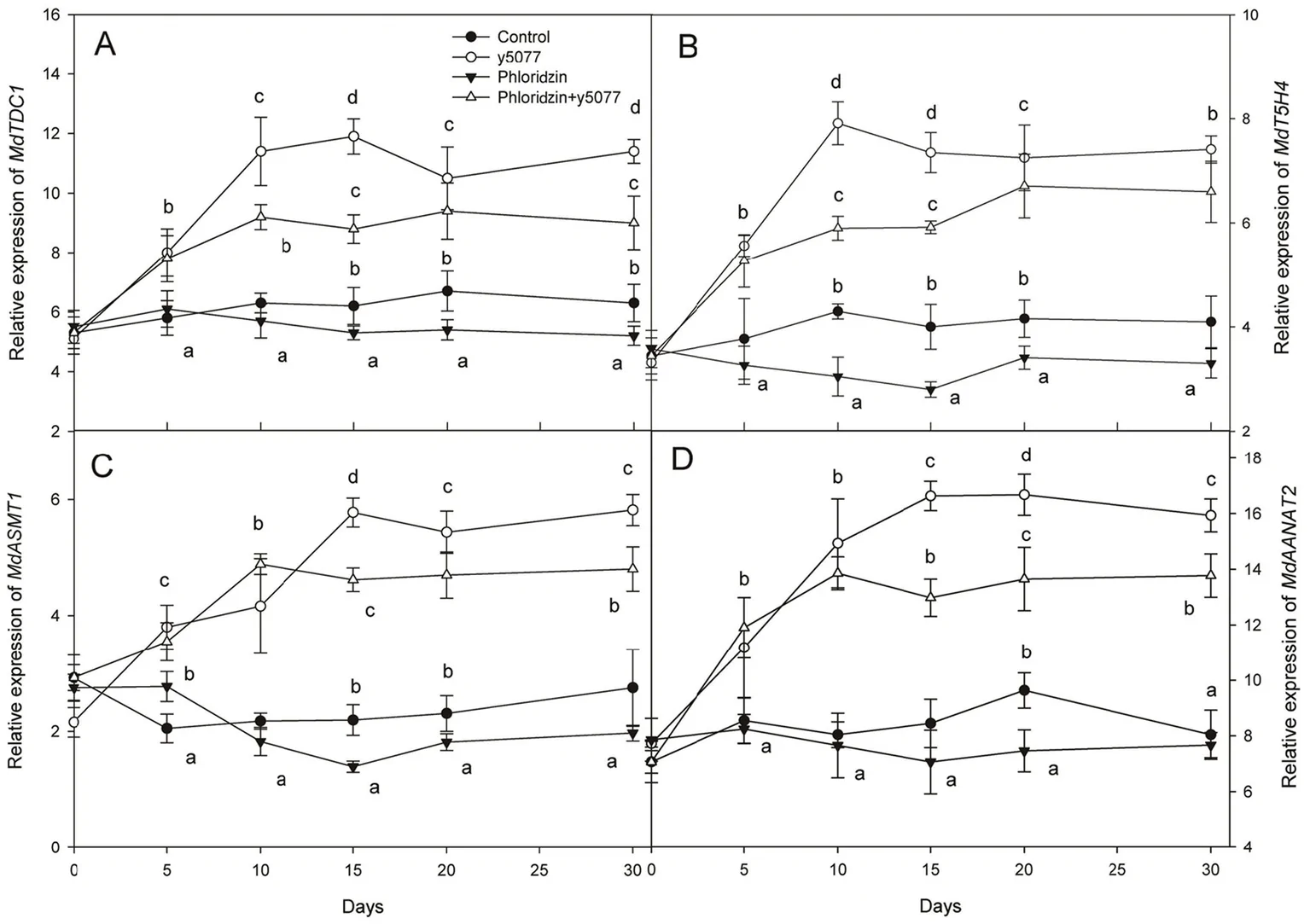
Effect of rhizobia inoculation on expression patterns of melatonin synthesis genes in *M. hupehensis* under phloridzin stress. **(A)** Expression of *MdTDC1*; **(B)** Expression of *MdT5H4*; **(C)** Expression of *MdAANAT2*; **(D)** Expression of *MdASMT1*. The total RNA was extracted from leaf after 2 h dark adaptation. Data are the means (SD) of three replicates. Different lowercase letters above the bars indicate statistically significant differences between treatments according to Tukey’s HSD test (*p* < 0.05).

The protective role of y5077 was further confirmed by the assessment of oxidative damage using DAB *in situ* staining (Figure 3; Supplementary Table S4). In the phloridzin-only group, a progressive accumulation of H_2_O_2_ was observed in the leaves (Figure 4B), with the staining intensity reaching Level V by day 30, indicating severe oxidative stress. In contrast, the phloridzin +y5077 group exhibited significantly lighter staining throughout the 30-day period, with the intensity maintained at Level II–III. This reduction in H_2_O_2_ accumulation was consistent with the decreased MDA content observed in y5077-inoculated plants (Figure 4C). These findings demonstrate that *E. meliloti* y5077 protects *M. hupehensis* from phloridzin-induced autotoxicity by promoting physical growth and effectively scavenging excessive reactive oxygen species (ROS) (Figure 5).

### 2.3 *E. meliloti* y5077 induces endogenous melatonin biosynthesis and enhances ROS scavenging

**Figure 6:**
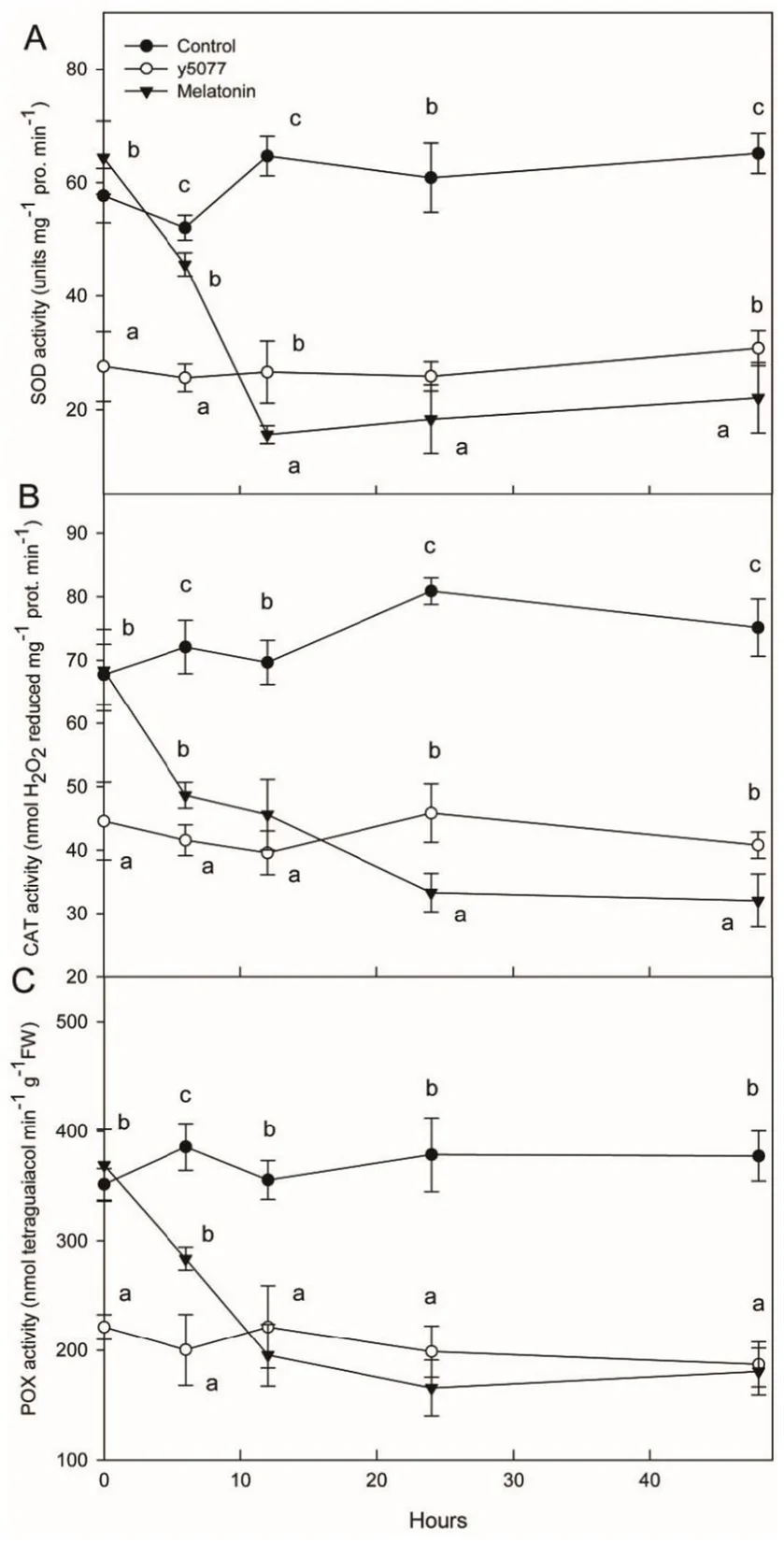
Effect of rhizobia inoculation and exogenous melatonin on the antioxidant enzymes activity in *M. hupehensis* plants under phloridzin stress. **(A)** SOD activity; **(B)** CAT activity; **(C)** POX activity. Two weeks old greenhouse seedling, with or without rhizobia inoculation, grown in 100 μmol kg^−1^ phloridzin were used in this experiment. Melatonin (1 mM melatonin in distilled water; Sigma) was sprayed on front and back of all leaves. Sampling started after 0, 6, 12, 24, and 48 of melatonin treatment. Data are the means (SD) of three replicates. Different lowercase letters above the bars indicate statistically significant differences between treatments according to Tukey’s HSD test (*p* < 0.05).

To elucidate the physiological mechanisms by which *E. meliloti* y5077 alleviates phloridzin-induced autotoxicity, we analyzed the changes in endogenous melatonin levels and the expression of genes involved in its biosynthesis and reactive oxygen species (ROS) scavenging. In the pot experiments, phloridzin stress initially triggered a modest increase in melatonin compared to the control. However, inoculation with y5077 significantly boosted these levels further. Two-way ANOVA revealed that endogenous melatonin levels were significantly influenced by Treatment (*F*_3, 24_ = 142.58, *p* < 0.001), Time (*F*_3, 24_ = 89.34, *p* < 0.001), and their interaction term (Treatment × Time: *F*_9, 24_ = 28.65, *p* < 0.001). Specifically, the melatonin concentration in the Phloridzin + y5077 group increased by 120, 160, 408, and 316% at 5, 10, 20, and 30 days, respectively, compared to the phloridzin group (Figure 4A). This surge in melatonin was consistently supported by the significant upregulation of key biosynthesis genes, including *MdT5H*, *MdSNAT*, *MdASMT*, and *MdCOMT*. Notably, at 20 days, the transcript levels of *MdSNAT* and *MdCOMT* in y5077-inoculated plants under phloridzin stress were 3.2-fold and 2.8-fold higher, respectively, than those in the phloridzin group (Figure 6).

The induction of melatonin biosynthesis effectively activated the plant’s enzymatic antioxidant defense system. SOD activity was elevated in seedlings in response to 100 μmol kg^−1^ phloridzin, reaching levels 2.2 times higher than those in the rhizobia-inoculated group. Interestingly, when exogenous melatonin (1 mM) was applied, SOD activity decreased gradually after 6 h and reduced to a level close to that of the rhizobia-inoculated plants after 12 h (Figure 6A). Similar trends were observed for CAT and POX activities, suggesting that y5077 improves *M. hupehensis* resistance by enhancing endogenous melatonin production to maintain ROS homeostasis (Figures 6B,C).

In addition to enzymatic scavenging, the non-enzymatic ascorbate-glutathione (AsA-GSH) cycle was recruited to prevent oxidative damage at the cellular level. Compared with the control group, the expression of key genes in this cycle—*MdcAPX*, *MdDHAR1*, *MdDHAR2*, and *MdcGR*—was markedly up-regulated after seedlings were transplanted into phloridzin-treated soil. However, rhizobia inoculation significantly abolished the excessive induction of these stress-responsive genes by phloridzin: 26% for MdcAPX, 54% for MdDHAR1, 51% for MdDHAR2, and 34% for MdcGR after 20 days of cultivation (Supplementary Figure S3).

These molecular and physiological responses were visually confirmed by DAB *in situ* staining of the leaves (Figure 3). While phloridzin treatment alone led to severe H_2_O_2_ accumulation reaching Level V (intense dark brown granulation) by day 30, the presence of y5077 effectively suppressed ROS levels, maintaining the staining at Level II–III throughout the experimental period. These results collectively indicate that *E. meliloti* y5077 enhances the autotoxicity resistance of *M. hupehensis* in association with increased endogenous melatonin levels and the coordinated activation of enzymatic and non-enzymatic ROS scavenging pathways.

### 2.4 *E. meliloti* y5077 improves soil enzymatic activities

**Figure 7:**
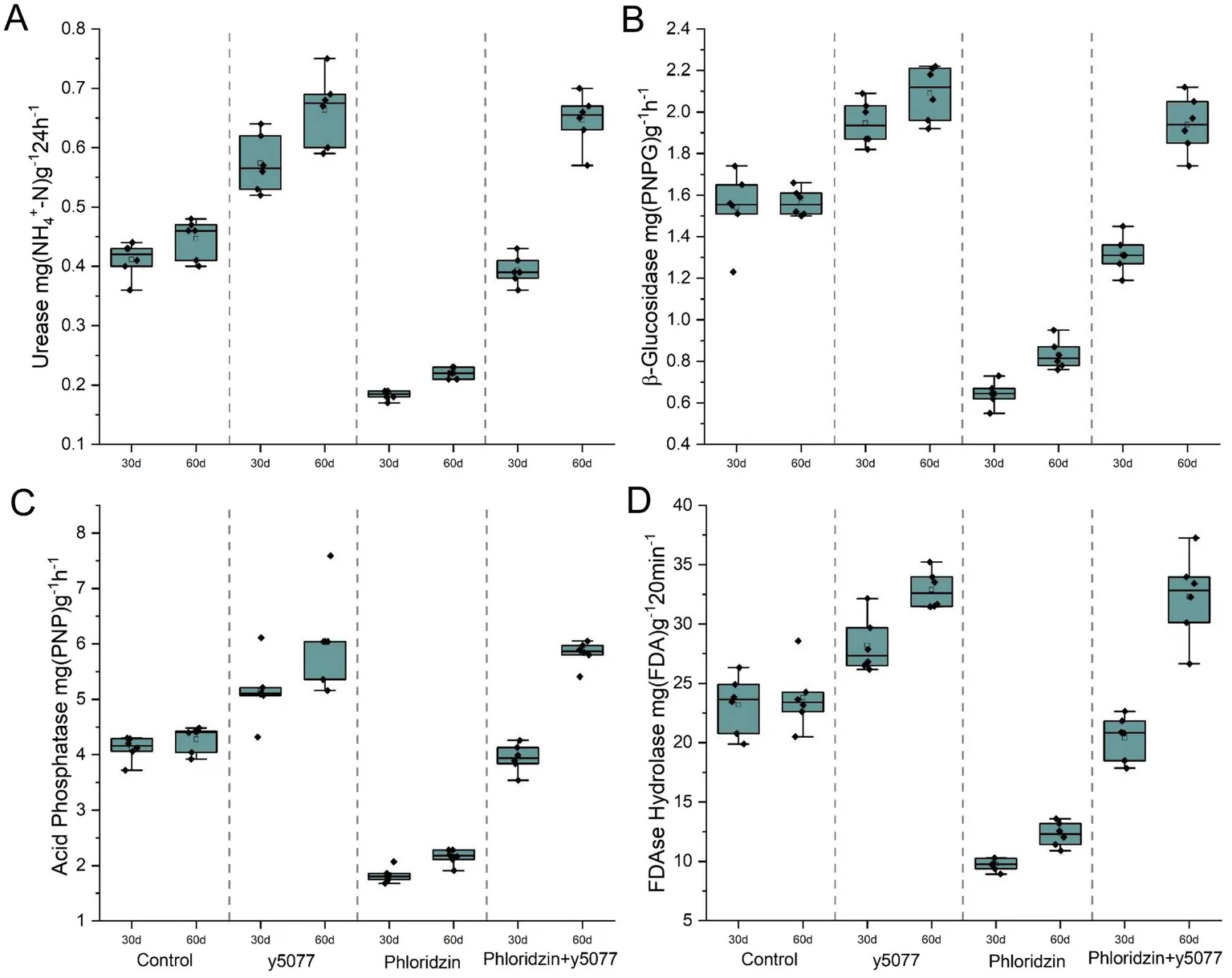
Effect of rhizobia inoculation on soil enzyme activities under phloridzin stress. **(A)** Urease activity, **(B)** *β*-glucosidase activity, **(C)** Acid Phosphatase activity, **(D)** FDAse activity. Boxplots display minimum, maximum, lower quartile, upper quartile, median, mean, and possible outliers. Data are mean ± SD (*n* = 6). Results of Two-way ANOVA: Urease (Treatment: *F*3,32 = 165.30, *p* < 0.001; Time: *F*4,32 = 112.15, *p* < 0.001; Treatment × Time: *F*12,3_2_ = 22.41, *p* < 0.001). Different letters indicate significant differences at *p* < 0.05 according to Tukey’s HSD post-hoc test.

Soil enzymatic activities are essential indicators of soil biochemical health, reflecting the intensity of nutrient cycling and microbial metabolism. As illustrated in Figure 7, phloridzin stress significantly suppressed the activities of all four measured enzymes compared to the control throughout the experimental period. By day 60, the activities of urease (nitrogen cycling), *β*-glucosidase activity (carbon cycling), acid phosphatase (phosphorus cycling), and FDA hydrolase (total microbial activity) in the phloridzin group had decreased by 65.1, 75.8, 53.7, and 73.1%, respectively, relative to the control. In contrast, inoculation with *E. meliloti* y5077 effectively reversed this inhibitory trend. By the end of the 60-day cultivation, enzyme activities in the phloridzin+y5077 group showed a robust recovery, reaching levels that were 3.77, 3.14, 4.05, and 3.54 times higher than those in the phloridzin group, respectively. Notably, the enzyme activities in the y5077-inoculated soil nearly returned to or even exceeded the baseline levels of the control. These findings demonstrate that *E. meliloti* y5077 alleviates phloridzin-induced biochemical barriers by restoring key enzymatic functions and promoting the overall metabolic vitality of the rhizosphere soil.

### 2.5 *E. meliloti* y5077 restructures the rhizosphere microbial community under phloridzin stress

To elucidate the biological mechanisms by which *E. meliloti* y5077 alleviates the replant-related autotoxicity of *M. hupehensis*, we comparatively analyzed the rhizosphere microbial communities under phloridzin stress and after restoration with y5077 inoculation. Bacterial 16S rRNA sequencing yielded 1,598,645 clean reads, while fungal ITS sequencing yielded 799,131 clean reads.

Alpha diversity analysis showed that y5077 inoculation effectively restored the bacterial richness and diversity inhibited by phloridzin (Supplementary Figure 3). Specifically, the bacterial Chao1 index significantly increased from 1062.42 ± 23.93 in the phloridzin group to 1130.73 ± 47.60 in the phloridzin+y5077 group (*p* = 0.0209). More notably, the bacterial Shannon index exhibited a highly significant increase, rising from 7.59 ± 0.14 to 8.13 ± 0.13 (*p* < 0.01), indicating a substantial improvement in community evenness and complexity. In contrast, the alpha diversity (Chao1 and Shannon indices) of the fungal community showed no significant difference between the phloridzin and phloridzin+y5077 groups (*p* > 0.05). In the fungal community, however, the addition of y5077 altered the original dominance distribution, shifting the overall fungal community composition. These results suggest that the restorative effect of y5077 was more pronounced in the bacterial portion of the microbiome than in the fungal portion (Supplementary Figures S4C,D).

**Figure 8:**
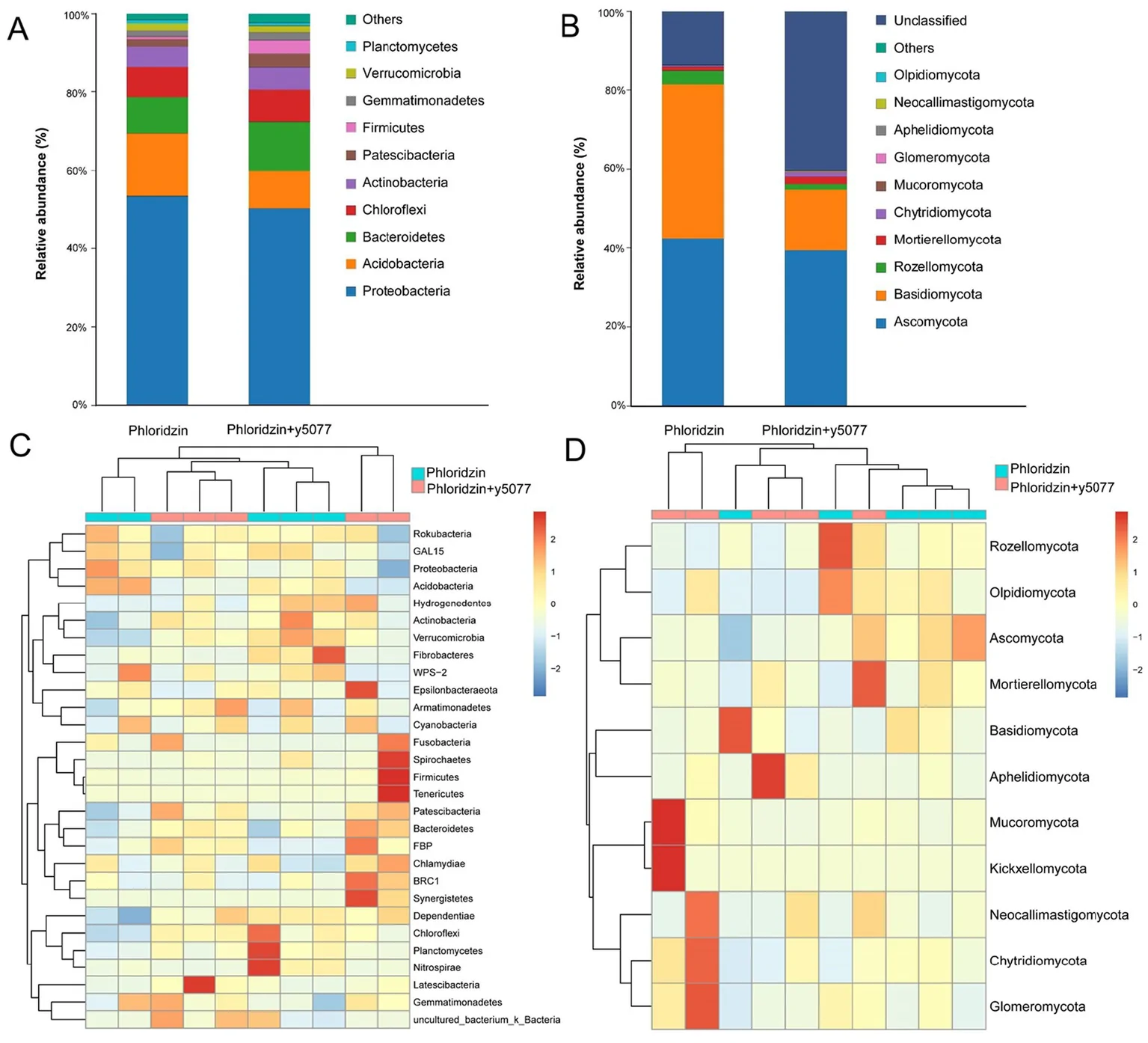
Taxonomic composition and relative abundance of rhizosphere microbial communities. Relative abundance of major taxa at the phylum levels for **(A–C)** bacterial phyla, **(B–D)** fungal phyla.

**Figure 9:**
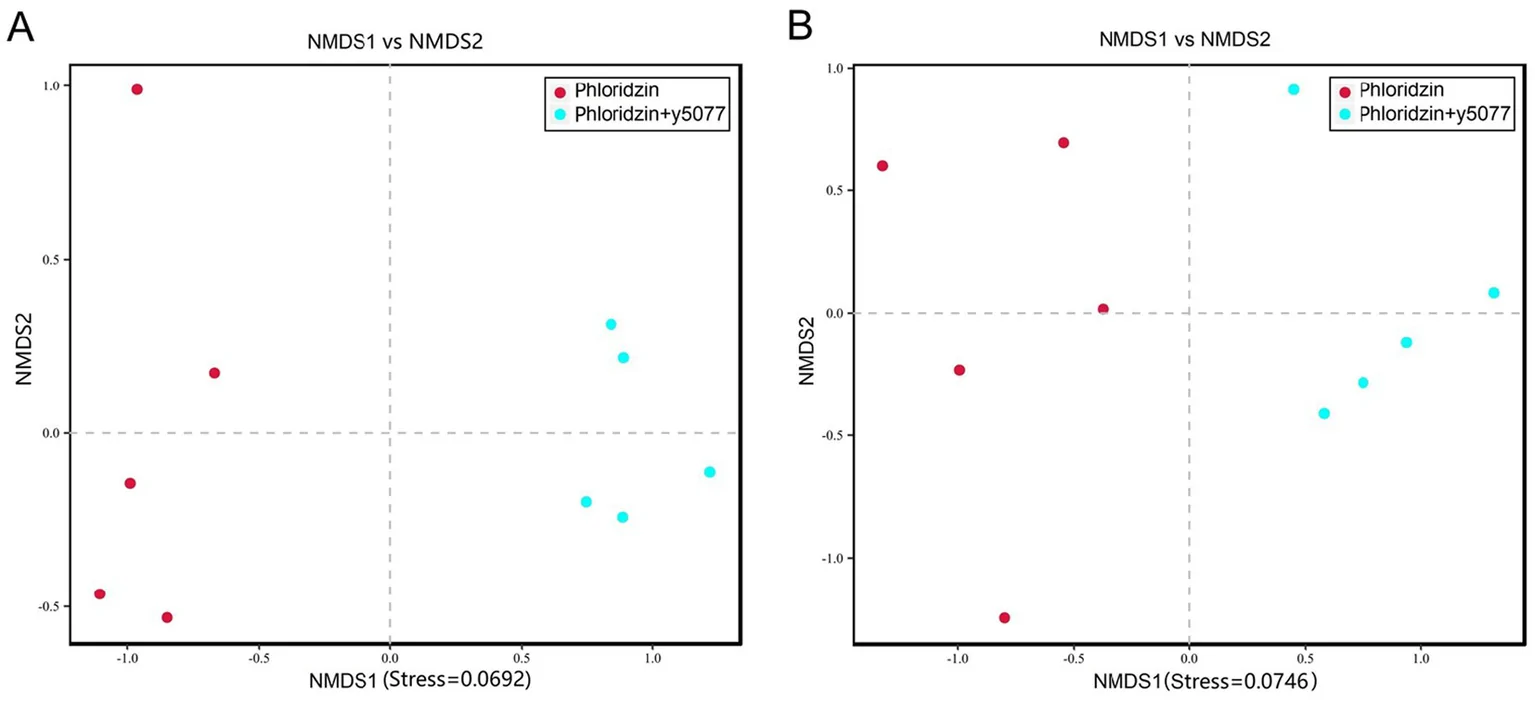
Non-metric multidimensional scaling (NMDS) analysis of rhizosphere microbial communities. NMDS plots showing the structural shifts in **(A)** bacterial communities (16S rRNA) and **(B)** fungal communities (ITS) based on the Bray-Curtis distance matrix. Each point represents an individual biological replicate (*n* = 5). The stress value indicated in each plot represents the degree of fit; a stress value < 0.2 indicates that the 2D configuration provides a reliable representation of the community distances. Differences in community structure between treatments were statistically significant as evaluated by PERMANOVA (Bacterial community: *R^2^* = 0.45, *p* = 0.008; Fungal community: *R*^2^ = 0.38, *p* = 0.012).

Taxonomic profiling at the phylum level revealed that Proteobacteria (53.72%) and Acidobacteria (19.12%) were the primary bacterial phyla, while Ascomycota (42.88%) was the dominant fungal phylum (classified) across all samples (Figure 8). At the phylum level, y5077 inoculation significantly restructured the rhizosphere bacterial composition (Figure 8C). Specifically, the relative abundances of Actinobacteriota and Firmicutes were markedly enhanced. Bacteroidetes, Chloroflexi, Planctomycetota, and Nitrospirota also exhibited higher relative abundances in the y5077-treated group compared to the phloridzin-only group. In contrast, the abundance of Acidobacteriota significantly declined following treatment. Regarding the fungal community (Figure 8D), Mortierellomycota showed a significant increase in the y5077 group, and the abundance of Ascomycota was notably higher than that observed under phloridzin stress alone. Additionally, Rozellomycota and Olpidiomycota exhibited an increasing trend. Conversely, the relative abundance of Basidiomycota was significantly suppressed after y5077 inoculation, with a concomitant decrease in the abundances of Mucoromycota and Glomeromycota. NMDS analysis further confirmed that the inoculation of *E. meliloti* y5077 induced a distinct shift in the overall community structure. The phloridzin and phloridzin+y5077 groups formed separate clusters in the bacterial and fungal NMDS spaces, respectively. PERMANOVA testing statistically validated these structural shifts for both the bacterial (*R^2^* = 0.45, *p* = 0.008) and fungal (*R^2^* = 0.38, *p* = 0.012) communities, demonstrating that strain y5077 effectively restructured the rhizosphere micro-ecosystem under phloridzin stress (Figure 9).

To establish definitive functional linkages between microbial eco-restoration and host physiological responses, a comprehensive Spearman’s rank correlation analysis was performed (Supplementary Figure S5). The relative abundance of the shifted bacterial phyla, Actinobacteriota and Firmicutes, which were significantly enriched following *E. meliloti* y5077 inoculation, exhibited strong and significant positive correlations with host endogenous melatonin levels and antioxidant enzyme activities (*p* < 0.01). Concurrently, these beneficial bacterial taxa displayed tightly coupled negative correlations with soil phloridzin residues and leaf MDA accumulation (*p* < 0.01). In stark contrast, the opportunistic fungal groups (Basidiomycota and Mucoromycota) that were suppressed by y5077 showed the exact opposite correlation trends, tracking positively with toxic chemical burdens and cellular lipid peroxidation. These statistically rigorous linkages clearly demonstrate that the targeted reassembly of the rhizosphere microbiome actively drives the holistic alleviation of host autotoxic stress.

## 3 Discussion

### 3.1 *E. meliloti* y5077 alleviates phloridzin-induced autotoxicity in *M. hupehensis* through degradation

Apple replant disease (ARD) is a complex micro-ecological phenomenon, in which phloridzin-mediated autotoxicity is recognized as a primary factor hindering apple root development. In this study, we demonstrated that inoculation with *E. meliloti* y5077 significantly mitigated the growth inhibition caused by phloridzin in *M. hupehensis* seedlings, as evidenced by a substantial recovery in both plant height and total biomass. Since the past decade, *Rhizobia* has been found to have the ability to degrade various organic pollutants in the environment, including aromatic hydrocarbons and highly resilient and toxic heterocyclic and chlorinated compounds (Xu et al., 2023; Sui et al., 2023). Hence, rhizobia might be exploited for soil bioremediation in an autotoxic apple orchard. Although it is believed that plant-derived phenolic acid can destruct the cell membranes and inhibit the activity of critical enzymes of bacteria (Khameneh et al., 2019), rhizobia have evolved mechanisms to counteract, nullify, and even utilize these allelopathic compounds in root exudates as nutrients along with their ability to establish plant-bacteria interactions (Ratu et al., 2022). In addition to the symbiosis between rhizobia and legumes—formation of root nodules—it is known that rhizobia can also colonize root surfaces using root exudates as nutrient sources and promote the growth of many non-legume (Sujkowska-Rybkowska et al., 2022). Our results showed that the ability of rhizobia to withstand the toxicity of phenolic acids was varying widely. And *E. meliloti* y5077 had the highest tolerance to phloridzin toxicity in this study (Supplementary Table S2). The levels of phloridzin we tested (50-200 μmol/kg) encompass both the average levels found in typical ARD soils and the extreme concentrations localized in rhizosphere micro-environments. The robust survival and degradative activity of *E. meliloti* y5077 under these conditions highlight its potential to neutralize the toxic “hotspots” that are primarily responsible for seedling growth inhibition in field conditions.

### 3.2 Elevated melatonin levels are associated with the activation of the antioxidant system to alleviate oxidative stress

There has been very little research reported on the protective effect of rhizobia against plant autotoxicity. In the present work, *E. meliloti* y5077 not only showed efficient degradation of phloridzin but also alleviated the oxidative stress caused by phloridzin. Numerous studies have shown phenolic acid compounds, including phloridzin, served as allelochemicals and can cause an imbalance of oxidation–reduction equilibrium in the recipient plants (Savina et al., 2023; Stryjecka et al., 2023). As a result, excessive production of ROS leads to local tissue necrosis or whole plant death. MDA is a major reactive aldehyde resulting from the peroxidation of biological membranes. It is usually used as an indicator of the degree of oxidative stress in plants (Wang et al., 2024). Plant scavenging mechanisms are needed to prevent the toxic effects of ROS to protect cells from the deleterious effects of allelochemicals (Sharma et al., 2022; Kim et al., 2017). Antioxidant enzymes (SOD, CAT, and POX) and non-enzymatic antioxidants both play important roles in scavenging ROS (Caverzan et al., 2016). The present study showed that rhizobia inoculation could alleviate oxidative stress by inducing melatonin production instead of inducing the antioxidant system of plants (Figures 4, 6). It should be noted that while transcript levels are strong indicators of systemic stress responses, they may not always perfectly translate to *in vivo* enzymatic activity due to complex post-transcriptional and post-translational modifications. However, the comprehensive phenotypic improvements observed in this study—specifically the significant reductions in MDA content and the visibly diminished ROS accumulation in root tissues—provide robust functional validation. These downstream physiological readouts confirm that the transcriptomic upregulation successfully translated into a substantially enhanced overall antioxidant capacity in *M. hupehensis* following y5077 colonization.

Melatonin is an amphipathic molecule that can function as an antioxidant signaling molecule under stress conditions (Back et al., 2016). Oxidative stress arises from an imbalance between ROS production and the detoxification of their reactive intermediates, which is of vital importance if plants are to maintain intracellular ROS pools at low levels. Various mechanisms control temporal and spatial coordination between ROS and other signals that are activated in separate parts of the plant at different times (Mittler et al., 2022). Among the ROS, H_2_O_2_ participates in a series of processes for plant development, stress responses, and programmed cell death (Petrov et al., 2015). We noted here that H_2_O_2_ content was significantly increased along with the decrease in melatonin production in phloridzin-treated seedlings. On the other hand, the melatonin content was increased significantly along with the decrease in H_2_O_2_ content in rhizobia-inoculated plants (Figure 4). Meanwhile, the exogenous application of melatonin was also associated with a reduction of oxidative stress in phloridzin-treated seedlings (Figure 6). Taken together, the results implied that melatonin might serve as a direct scavenger of free radicals and through a scavenging cascade (Galano et al., 2013).

**Figure 10:**
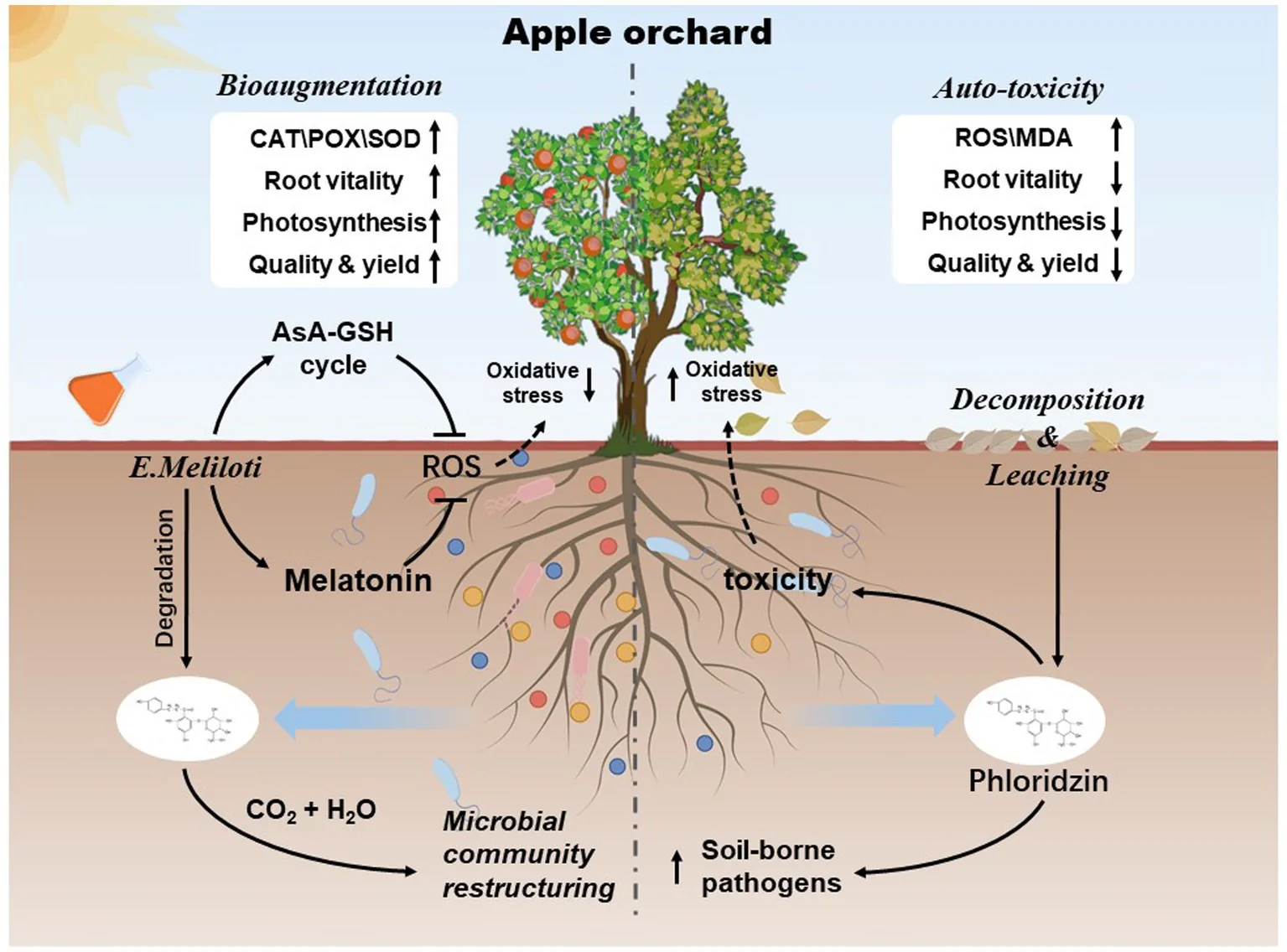
A proposed conceptual model illustrating the multi-functional mechanisms of *Ensifer meliloti* y5077 in mitigating phloridzin-induced autotoxicity in *Malus hupehensis*. The bioaugmentation process is systematically delineated into three interconnected logical levels: (1) Rhizosphere Detoxification: *E. meliloti* y5077 efficiently colonizes the root surface and degrades the accumulated autotoxin phloridzin into harmless organic remnants; (2) Physiological and enzymatic regulation: The mitigation of chemical stress prompts the restoration of host endogenous melatonin levels and coordinates the homeostatic balancing of antioxidant enzyme cascades (SOD, POX, and CAT), systematically suppressing reactive oxygen species (ROS) and malondialdehyde (MDA) accumulation; and (3) Ecological Rebalancing: The alleviated autotoxic burden effectively restructures the rhizosphere microbiome. Standardized arrows and flathead lines denote activation and suppression pathways, respectively.

It is noteworthy that a small portion of phloridzin in spiked soil cannot be effectively degraded by rhizobia with the presence of seedlings (Supplementary Table S1). The concentrations of undegraded phloridzin (below 15 μmol kg^−1^) are close to that in the soil of the orchard with the ARD problem (Wang et al., 2015). It might have a toxic effect on the root system of *M. hupehensis* seedlings as shown in Supplementary Table S3. Therefore, the protective effects of rhizobia against autotoxicity consist of the removal of phloridzin in the rhizospheric soil and reducing the oxidative stress caused by phloridzin in the tissue internally. The two protective processes of applying rhizobia to apple seedlings seem to be independent of each other (Figure 10).

While our findings demonstrate that *E. meliloti* y5077 colonization significantly elevates endogenous melatonin levels in *M. hupehensis*, the exact regulatory cascade warrants further exploration. This elevation is likely driven by a synergistic combination of indirect stress relief and direct microbial signaling. Indirectly, the rapid degradation of phloridzin by y5077 drastically reduces the primary source of oxidative stress in the rhizosphere. The consequent alleviation of the internal ROS burden allows the plant to restore its intrinsic metabolic functions, including melatonin biosynthesis, which is typically suppressed under severe autotoxicity. Concurrently, as a typical plant growth-promoting rhizobacterium (PGPR), *Ensifer* species can secrete microbe-associated molecular patterns (MAMPs) or other signaling molecules that directly elicit induced systemic resistance (ISR), thereby actively upregulating plant hormone networks. Here, we link y5077 to a protective phenotype, but causal involvement of the melatonin pathway remains unproven due to the absence of loss-of-function data. Future studies utilizing specific rhizobial mutants (e.g., phloridzin-degradation deficient strains) and multi-omics approaches will be essential to completely disentangle these direct and indirect pathways.

### 3.3 Systemic restructuring of the soil micro-environment and microbial community stability

Soil enzymatic activities are essential indicators of biochemical health and nutrient cycling efficiency within the rhizosphere ecosystem. In the present study, phloridzin stress was found to significantly suppress the activities of four key enzymes—urease, *β*-glucosidase, acid phosphatase, and FDA hydrolase—under a neutral soil pH of 7.2. This widespread inhibition suggests a fundamental disruption in the carbon, nitrogen, and phosphorus cycles, which is a characteristic feature of soil degradation associated with continuous monocropping. However, the inoculation of *E. meliloti* y5077 effectively reversed this inhibitory trend. By the end of the 60-day cultivation period, the activities of these enzymes in the y5077-inoculated soil had significantly increased, reaching levels higher than those in the phloridzin-only group and nearly returning to or even exceeding the baseline levels of the control.

Interestingly, soil enzyme activities in the y5077-inoculated treatments often surpassed those observed in the non-stressed Control group (Figure 9). This “super-recovery” phenomenon highlights a robust bioaugmentation effect. The high colonization density of y5077 directly enriches the extracellular enzyme pool through its own metabolic secretion. Simultaneously, the alleviation of autotoxicity allows the roots to restore their normal exudation patterns, providing carbon sources that further stimulate the metabolic turnover of both the inoculum and recruited beneficial microbes (e.g., Firmicutes). Consequently, the elevated enzyme activities reflect a significant enhancement of the rhizosphere’s functional capacity in nutrient cycling, rather than a transient spike, providing a healthier biological foundation for apple seedling growth.

This biochemical recovery was closely associated with a fundamental restructuring of the rhizosphere microbial community. High-throughput sequencing results revealed that the bacterial component of the microbiome was particularly responsive to y5077 inoculation. The significant increases in both the bacterial Chao1 and Shannon indices (*p* < 0.05 and *p* < 0.01, respectively) indicate that strain y5077 not only restored species richness but also enhanced community evenness and potential functional redundancy. Such improvements in diversity are crucial for maintaining the resilience of the soil ecosystem against allelochemical stress. While the fungal community’s alpha diversity remained relatively stable, NMDS analysis demonstrated a significant structural shift in both bacterial and fungal communities. The microbial community reassembly under phloridzin stress conditions is characterized by significant shifts in the relative abundance of specific bacterial and fungal taxa. The significant increases in Actinobacteriota and Firmicutes, along with the marked reduction in Basidiomycota and Mucoromycota, indicate a major structural shift. While these phyla encompass members with highly diverse ecological roles, their broad compositional changes reflect a distinct microbial response to the alleviation of autotoxic stress, though genus-level or functional analyses are required to define their specific ecological functions (Somera and Mazzola, 2022; Demyanyuk et al., 2025). While the phylum *Basidiomycota* generally comprises vital saprophytic and mutualistic fungi essential for soil health, its decline in the specific context of ARD may represent a positive indicator of root recovery. Under severe autotoxicity, the accumulation of necrotic root tissues often selectively enriches opportunistic decay *Basidiomycetes*, which can exacerbate root rot. The reduction of this phylum following y5077 inoculation likely reflects the alleviation of root necrosis and the subsequent withdrawal of these opportunistic degraders. Furthermore, the potential gap in saprophytic nutrient cycling is likely compensated by the robust enrichment of *Mortierellomycota*, a fungal group widely recognized for its rapid organic matter decomposition and pathogen-antagonizing capabilities. Nonetheless, as general basidiomycete populations are suppressed, long-term field monitoring is advisable to ensure that the broad recalcitrant carbon cycling functions of the soil are not unintentionally impaired.

Furthermore, the high colonization density of *E. meliloti* y5077 (10^7^ CFU/g) indicates that phloridzin likely acts as a potent chemoattractant, driving its targeted recruitment. While the introduction of a dominant exogenous strain might raise concerns regarding indigenous ecological disruption, this bioaugmentation process is inherently substrate-dependent. The transient abundance of y5077 is sustained by the acute phloridzin toxicity; as the autotoxin is progressively degraded, this specific niche advantage diminishes. Consequently, the exogenous strain naturally integrates into the native microbiome without causing permanent ecological displacement. Ultimately, rather than disrupting the indigenous ecology, y5077 successfully steered the disturbed microbial community toward a more resilient and balanced state. Although current 16S rRNA sequencing provides primarily correlative evidence, these specific taxonomic shifts collectively support the functional recovery of the soil ecosystem, while the definitive establishment of metabolic causality awaits further validation through future meta-transcriptomic studies.

The conclusion that the rhizosphere microbiome underwent significant community reassembly following y5077 inoculation is supported by three converging lines of direct evidence. First, the significant elevation of the bacterial Shannon index reflects enhanced community evenness and complexity, which are widely recognized as key determinants of ecological resilience against stress. Second, taxonomic rebalancing occurred through the significant increases in the relative abundance of specific taxa (e.g., *Firmicutes*, *Actinobacteriota*, and *Mortierellomycota*) and the concurrent reduction of others (e.g., *Basidiomycota* and *Mucoromycota*). Finally, this structural stability translated into functional stability, as evidenced by the comprehensive recovery of soil enzyme activities, demonstrating that the reassembled microbial community effectively supports robust biogeochemical cycling.

However, it is important to acknowledge the limitations of our experimental setup. While the greenhouse pots utilized soil with an indigenous microbiome, amending it with a single exogenous allelochemical (phloridzin) represents a highly simplified simulation of Apple Replant Disease (ARD). Future studies using authentic orchard replant soils are required to fully evaluate the bioaugmentation efficacy of *E. meliloti* y5077.

## 4 Conclusion

In summary, phloridzin-induced autotoxicity markedly inhibits the biomass accumulation and photosynthetic efficiency of *M. hupehensis*. However, inoculation with *E. meliloti* y5077 effectively mitigates these negative impacts and restores plant growth. This protective mechanism is driven by a dual strategy: the direct degradation of phloridzin at the soil-root interface, which is associated with the stimulation of an endogenous melatonin-related antioxidant defense system (Figure 10).

The findings underscore that specialized rhizobia represent a valuable biological resource for degrading phenolic autotoxins while providing inherent probiotic benefits to the host. While this study confirms the robust early-stage efficacy of *E. meliloti* y5077 in mitigating phloridzin-induced autotoxicity, transitioning to complex, multi-year field environments remains a future challenge. To counteract the continuous exudation of root autotoxins in orchards, future strategies should focus on developing slow-release bio-formulations and optimizing multi-frequency inoculation regimes. These approaches will be essential to sustain the functional microbial population and achieve long-term ecosystem stability.

It should be noted that this experimental design has a limitation: in natural soil, it is difficult to distinguish between the direct interactions (such as competition) of y5077 with native microorganisms and the indirect effects resulting from improved plant health. The alleviation of phloridzin autotoxicity alters root exudates, which in turn drives the restructuring of the microbiome. Future studies need to employ gnotobiotic systems, synthetic microbial communities, or genetic mutants to dissect these two types of mechanisms.

## 5 Materials and methods

### 5.1 Soil preparation

Soil samples were collected from the campus of the apple orchard in Yantai, Shandong, China, where the 5-year average precipitation is 650 mm, the annual average temperature is 12.7 °C, and the altitude is 100 ~ 920 m. The soil was air-dried for 2 weeks; then larger debris was removed by a sieve with a 2 mm aperture, retaining the indigenous microbial community baseline for subsequent analysis except phloridzin-degrading assay. Finally, the soil was autoclaved (121 °C, 15 min) to exclude interference from native microbiota and examine the direct phloridzin-degrading activity and colonization of rhizobia strains. Then the physical and chemical properties of soil were analyzed (Supplementary Table S5). Soil Texture: The percentages of sand, silt, and clay were determined to characterize the soil type. pH Measurement: Soil pH was measured in a 1:1 (w/v) soil-to-water suspension. Nutrient and Organic Matter: Inorganic nitrogen (NO_3_-N and NH_4_-N), available phosphorus (P), and organic matter content were analyzed to establish the baseline nutritional status of the soil. Field survey data indicate that its average concentration is 12.46 mg/kg, with peak levels reaching up to 27.13 mg/kg (approximately 62.2 μmol/kg) (Somera and Mazzola, 2022; Xiang et al., 2021). To simulate the rhizosphere “hotspots” formed during the root decomposition process in apple orchard soils and to establish a rapid acute stress experimental model, this study applied phloridzin to the soil at concentrations up to 200 μmol/kg. Prior to addition, the phloridzin solution was sterilized by passing it through a 0.22 μm filter, and then used to prepare soil treatment groups across four concentration gradients: 50, 100, 150, and 200 μmol/kg.

### 5.2 Rhizobia isolation and plant cultivation

Rhizobia strains were originally isolated from legume plants in a saline soil area (salt content 0.3–0.7%) of the Yellow River Delta, Shandong, China (37.77°N, 118.97°E). A total of 119 isolates were obtained. All isolates were subjected to molecular identification and phylogenetic analysis using housekeeping gene *recA* and 16S rDNA as conserved sequences, respectively, as in our previous work (Ren et al., 2016). To screen for strains capable of using phloridzin as the sole carbon source, isolates were propagated on basal mineral agar plates (NH_4_Cl, K_2_HPO_4_, KH_2_PO_4_, MgSO_4_, pH 6.5) with 10 μM phloridzin (Sigma; St. Louis, MO, USA) at room temperature. Four isolates (*Ensifer meliloti* y4071, *Ensifer meliloti* y5077, *Ensifer arboris* y4009, and *Ensifer arboris* y4031) that can grow on a screening medium were selected for follow-up experiments. Phloridzin degradation by the isolates was performed three times in a liquid basal mineral medium with 10, 20, 40, and 60 mM phloridzin, respectively.

After stratification at 4 °C for 1 month, *M. hupehensis* seeds were surface-sterilized before use. After 2 weeks of growth, every three uniform seedlings (5–6 leaves) were transferred into a single 5-L pot with prepared soil as one replicate. To ensure true biological replication and avoid pseudo-replication, a single pot was strictly defined as one independent experimental unit throughout the study. For physiological and biochemical evaluations, three independent pots were randomly selected per treatment (*n* = 3). All the pots were arranged randomly in a climate chamber with day/night temperatures of 28/20 °C, 50 ± 2% relative humidity, and a 12/12 h light/dark cycle. Rhizobia broth (0.8 × 10^9^ CFU mL^−1^) was used as an inoculant; 1 mL of inoculant was sprayed into 1 kg of soil. For time-course physiological, biochemical, and enzymatic measurements, independent pots were randomly selected and destructively harvested for each specific time point and treatment to avoid resampling stress.

After 4 weeks of cultivation, the *M. hupehensis* root was harvested to evaluate bacterial colonization. Root systems were rinsed gently to remove all attached debris. Then it was placed in a 100 mL tube containing 0.1% peptone and 0.2% Na-hexametaphosphate in deionized water and shaken vigorously for 10 min at 220 rpm. The suspensions were serially diluted and spread on basal mineral medium agar plates to determine the number of viable cells (cfu g^−1^ fresh weight). To ensure experimental rigor and verify strain identity, a qualitative verification protocol was conducted alongside enumeration. Ten colonies were randomly picked from the highest-dilution plates of each treatment group and subjected to colony PCR targeting the strain-specific recA gene (Suzuki et al., 2017). The results were compared with the recA gene sequence of each strain to determine the presence of rhizobia. Primers for recA gene amplification are 41F, 5‘-TTC GGC AAG GGM TCG RTS ATG-3’ and 640R, 5‘-ACA TSA CRC CGA TCT TCA TGC-3’ (Li et al., 2015).

### 5.3 Biomass and root architecture

After 10 weeks of cultivation, the *M. hupehensis* seedlings were measured for photosystem II efficiency (ΦPSII) and non-photochemical quenching of chlorophyll fluorescence (NPQ) as previously described (Kong et al., 2017). The plants were oven-dried and weighed to record the biomass. The roots were rinsed to remove all attached debris. After a thorough cleaning, the root system was arranged and scanned. The images of the root system were analyzed using the WinRHIZOR image system V4.1c (Regent Instruments, Quebec, Canada) for total lengths, surface area, volume, and the number of root tips and forks.

### 5.4 HPLC analysis of phloridzin and melatonin

For the phloridzin assay, 10 g of soil was extracted using 90% (v/v) ethanol at room temperature. After centrifugation, the supernatant was filtered with a 0.45 μm filter, lyophilized, and then dissolved in acidified methanol (0.2 M acetic acid) for analysis. The detection method was based on and modified from the work of Mindaugas et al. (Liaudanskas et al., 2014) The HPLC system (1,200, Agilent, Santa Clara, USA) was equipped with a Hypersil-ODS C18 column (4.6 mm × 250 mm, 5 μm; Thermo Fisher Scientific Inc., Waltham, MA). The mobile phase was 30% methanol and 70% 0.15 M acetic acid; the flow rate was at 0.8 mL min^−1^. The detector wavelength was at 286 nm, and the temperature was 28 °C. Each sample was replicated three times. Phloridzin was identified by comparison with the standards. The retention time was 7.14 min (Supplementary Table S6).

For the melatonin assay, 1 g of fresh leaf tissues was collected from seedlings after dark adaptation for 2 h and homogenized in pre-cooled 80% (v/v) methanol. After centrifugation at 5000 g for 5 min, the supernatant was filtered with a 0.45 μm membrane. Melatonin content was analyzed using the same HPLC system as above with an isocratic elution of 0.15 M acetic acid and methanol (60:40, v:v); the flow rate was at 0.9 mL min^−1^. The detector wavelength was at 220 nm, and the temperature was 30 °C. Each sample was replicated three times. Melatonin was identified by comparison with the standards (Sigma; St. Louis, MO, United States). The retention time was 3.53 min (Supplementary Table S6). The detection method was based on and modified from the work of Martins et al. (Martins et al., 2017).

### 5.5 Measurements of H_2_O_2_ and lipid peroxidation

Fresh leaf tissue was homogenized with 5% (w/v) trichloroacetic acid on ice. The H_2_O_2_ was measured as described by the method of Brennan and Frenkel (1977). One unit of H_2_O_2_ was defined as the chemiluminescence caused by the internal standard of 1 μM H_2_O_2_ g^−1^ fresh weight. Lipid peroxidation was determined by measuring malondialdehyde (MDA) using the thiobarbituric acid reaction as previously described (Ren et al., 2016). Leaf tissue was homogenized in 0.1% trichloroacetic acid on ice. After ultra-speed centrifugation, the supernatant was reacted with thiobarbituric acid. The MDA concentration was calculated based on the extinction coefficient, ɛ = 155 mM^−1^ cm^−1^.

### 5.6 Scanning electron microscopy (SEM) analysis

To observe the colonization of *Ensifer meliloti* y5077 on the root surface and within the root tissues of *M. hupehensis*, root segments (approximately 5 μm in length) were collected after 4 weeks of treatment and immediately fixed in 2.5% (v/v) glutaraldehyde at 4 °C for 12 h. The samples were then rinsed with 0.1 M phosphate-buffered saline (pH 7.2) and dehydrated through a graded ethanol series (30, 50, 70, 80, 90, and 100%). After critical point drying, the specimens were mounted on aluminum stubs with double-sided conductive carbon tape. Some segments were longitudinally fractured to examine internal colonization, and all specimens were sputter-coated with a thin gold–palladium layer to enhance conductivity. Observations were performed using a Hitachi SU8100 scanning electron microscope at an accelerating voltage of 3.00 kV. Micrographs were captured at x2.50k magnification (scale bar = 20.0 μm) to survey the bacterial distribution on the root system and at x10.0 k magnification (scale bar = 5.00 μm) to characterize the morphology and attachment details of individual cells.

### 5.7 Measurement of soil enzymatic activities, plant antioxidant enzymes activity and DAB staining

Soil enzymatic activities were determined following previous methods (Guo et al., 2024), six biological replicates were used per treatment. Urease (EC 3.5.1.5): Activity was measured using indophenol colorimetry. Briefly, 5 g of fresh soil was incubated with 10 mL of 10% (w/v) urea and 20 mL of citrate buffer (pH 6.7) at 37 °C for 24 h. The released ammonium was reacted with sodium phenoxide and sodium hypochlorite, and the absorbance was measured at 578 nm *β*-Glucosidase (EC 3.2.1.21): 1 g of fresh soil was mixed with 1 mL of 0.025 M p-nitrophenyl-*β*-D-glucopyranoside (PNPG) and 4 mL of modified universal buffer (MUB, pH 6.0). After incubation at 37 °C for 1 h, the reaction was terminated by adding 0.5 M CaCl_2_ and 0.1 M Tris-NaOH (pH 12.0), followed by spectrophotometric measurement at 400 nm. Acid Phosphatase (EC 3.1.3.2): This was assayed based on the release of p-nitrophenyl (PNP). 1 g of soil was incubated with 1 mL of 0.115 M p-nitrophenyl phosphate and 4 mL of MUB (pH 6.5) at 37 °C for 1 h. The reaction was stopped with 0.5 M CaCl_2_ and 0.5 M NaOH, and the absorbance was recorded at 420 nm. Fluorescein Diacetate (FDA) Hydrolase: Activity was determined by measuring the hydrolytic cleavage of FDA into fluorescein. 5 g of soil was mixed with 15 mL of 60 mM potassium phosphate buffer (pH 7.6) and 0.2 mL of 1,000 μg/mL FDA, then incubated at 30 °C with shaking (200 rpm) for 20 min. After adding a 2:1 chloroform/methanol solution to stop the reaction, the mixture was centrifuged, and the supernatant was measured at 490 nm.

One millimole per liter of melatonin (Sigma; St. Louis, MO, United States) solution was sprayed on the front and back of all leaves. Fresh leaf tissues were homogenized with a buffer solution containing 1% (w/v) polyvinylpolypyrrolidone, 50 mM potassium phosphate, pH 7.8, and 1 mM EDTA-Na_2_ on ice. Superoxide dismutase (SOD; E. C. 1.15.1.1), catalase (CAT; E. C. 1.11.1.6), and peroxidase (POX; E. C. 1.11.1.7) activities were measured separately according to our previous work (Ren et al., 2016). The sampling method is described above. Each sample had 3 plants and was in triplicate.

*In situ* accumulation of H_2_O_2_ in leaf tissues was visualized using 3,3′-diaminobenzidine (DAB) staining following previous work (Ren et al., 2014). Pretreated seedling leaves were sectioned into 1 cm fragments and vacuum-infiltrated for 30 min in a solution containing 0.5 mg mL^−1^ DAB and 0.01% (v/v) Triton X-100. The presence of H_2_O_2_ was identified by the formation of brown polymerization products, and the experiment was performed in triplicate (using leaves collected from three independent pots as true biological replicates) to ensure consistency. The degree of staining was qualitatively categorized into five levels and scored blindly by an independent observer to prevent bias: level I (no pigment deposition), level II (slight brown deposition), level III (heavy brown deposition), level IV (entire leaf stained brown), and level V (localized intense brown-black staining). Furthermore, ImageJ software (NIH, Bethesda, MD, United States) was utilized for the quantitative analysis of the brown polymerized areas to statistically support the visual qualitative evaluations.

### 5.8 High-throughput sequencing

Total genomic DNA was extracted from 0.5 g of rhizosphere soil samples using the FastDNA Spin Kit for Soil (MP Biomedicals, United States) according to the manufacturer’s protocols. The quality and concentration of DNA were assessed via 1.0% agarose gel electrophoresis and a NanoDrop 2000 spectrophotometer (Thermo Fisher Scientific, United States). For the high-throughput sequencing of the rhizosphere microbiome, five independent pots were randomly selected per treatment (*n* = 5), and the rhizosphere soil from each selected pot was collected and processed as a distinct biological replicate.

For bacterial community analysis, the V3–V4 hypervariable region of the 16S rRNA gene was amplified using the universal primers 338F (5’-ACTCCTACGGGAGGCAGCAG-3′) and 806R (5’-GGACTACHVGGGTWTCTAAT-3′). For fungal community analysis, the ITS1 region was amplified using the primers ITS1F (5’-CTTGGTCATTTAGAGGAAGTAA-3′) and ITS2R (5’-GCTGCGTTCTTCATCGATGC-3′). The PCR reactions were performed in triplicate to ensure reliability. Each reaction was carried out in a 20 *μ*L mixture containing 4 μ L of 5 × FastPfu Buffer, 2 μL of 2.5 μM dNTPs, 0.8 μL of each primer (5 μM), 0.4 μL of FastPfu Polymerase, and 10 ng of template DNA.

*Library preparation and sequencing*: Following amplification, the resulting PCR products were purified using the AxyPrep DNA Gel Extraction Kit (Axygen Biosciences, United States) and quantified with a QuantiFluor™-ST Blue Fluorescence Quantitative System (Promega, United States). Purified amplicons were pooled in equimolar amounts and paired-end sequenced (2 × 250 bp) on the Illumina PE250 platform (Illumina, San Diego, USA) according to standard protocols. Raw data was submitted to the China National Center for Bioinformation with the project number PRJCA059406.

### 5.9 Quantitative real-time PCR

Gene expression of enzymes involved in the ascorbate-glutathione cycle or melatonin synthesis was analyzed with quantitative real-time PCR (qRT-PCR). All primers used in this work are listed (Supplementary Table S7). Total RNA was extracted from fresh leaves using TRIzol (Invitrogen, Carlsbad, CA, USA). Three bio-replicates were prepared from each treatment. The cDNA was synthesized using the GoScript™ kit (Promega, Madison, WI, USA). The qRT-PCR was performed using the iCycler iQ Real-time PCR Detection System (Bio-Rad). The reaction system comprised 0.2 μM primers, 1 μL template cDNA, and 10 μL SYBR Green MasterMix (TaKaRa Bio Inc., Dalian, China). The program was 95 °C for 20 s; 32 cycles of 95 °C for 5 s, 58 °C for 10 s, and 72 °C for 28 s. *Malus* EF-1*a* gene (GenBank: DQ341381) was used as the internal control to standardize expression levels using the 2^−ΔΔCt^ method. The sampling method is described above. Each sample had 3 plants and was in triplicate.

### 5.10 Bioinformatics and statistical analysis

Raw sequencing reads were demultiplexed, quality-filtered using fastp (version 0.19.6), and merged by FLASH (version 1.2.11). Operational taxonomic units (OTUs) were clustered at a 97% similarity threshold using UPARSE (version 7.1), and chimeric sequences were identified and removed. Taxonomic assignment was performed using the RDP Classifier (version 2.11) against the Silva database (Release 138) for bacteria and the UNITE database (Release 8.0) for fungi, with a confidence threshold of 0.7. Alpha diversity indices, including Chao1, Shannon, and Simpson, were calculated to evaluate microbial richness and evenness. Beta diversity was evaluated using Principal Coordinate Analysis (PCoA) based on Bray-Curtis distance matrices. Differences in microbial/bacterial community structures among treatments were statistically tested using Permutational Multivariate Analysis of Variance (PERMANOVA) with 999 permutations (via the adonis2 function in the vegan R package).

To establish definitive functional linkages between microbial ecology and host plant responses, a Spearman’s rank correlation analysis was conducted to evaluate the relationships between the relative abundances of key rhizosphere microbial taxa and host physiological/chemical parameters. Crucially, all correlation coefficients (ρ) and statistical significances were calculated using individual pot-level data (*n* = 5 biological replicates per treatment group; total *N* = 20 or total sample size across groups) rather than treatment means to avoid standard error distortion and artificial inflation of correlation metrics. The correlation matrix and corresponding heatmap were constructed and visualized using the seaborn and matplotlib packages in Python.

For time-course physiological, biochemical, and soil enzymatic data, a Two-way Analysis of Variance (ANOVA) was conducted to evaluate the main effects of Treatment (T), Time (t), and their interaction (Treatment × Time, T × t). The exact degrees of freedom (d*f*_effect_, d*f*_error_), *F*-values, and *p*-values for all main effects and interaction terms are reported in the text and figure legends. Means were compared using Tukey’s HSD post-hoc test at *p* < 0.05. For single time-point measurements (e.g., plant biomass and alpha diversity indices), data were analyzed using a One-way ANOVA. When significant effects were detected, differences among treatment means were further evaluated using Tukey’s Honestly Significant Difference (HSD) post-hoc test at a significance level of *p* < 0.05. Statistical analyses were performed using SPSS v.19.0 (IBM, Inc., Armonk, NY, United States).

## Data Availability

The datasets presented in this study can be found in online repositories. The names of the repository/repositories and accession number(s) can be found in the article/Supplementary material.
