# Supplementary material for: Rhizobia mitigates autotoxicity in Malus hupehensis via phenolic degradation and enhanced melatonin-antioxidant responses
Source: Front Microbiol. 2026 Aug 13;17:1912880. doi: 10.3389/fmicb.2026.1912880 (PMC13518579; doi:10.3389/fmicb.2026.1912880)
Supplement: Supplementary file 1 [file Data_Sheet_1.docx]

**Supplementary information：**

*Rhizobia* Mitigates Autotoxicity in *Malus hupehensis* via Phenolic Degradation and Enhanced Melatonin-Antioxidant Responses

Supplementary table 1. The concentration of phloridzin in the soil with or without rhizobia treatment after 10 weeks.

|  | Initial concentration μmol kg^-1^ | | |  | |  | |  |
| --- | --- | --- | --- | --- | --- | --- | --- | --- |
|  | 0 | 50 | 100 | | 150 | | 200 | |
| Control | 0 | 47±6.1 | 95±8.5 | | 138±4.4 | | 158±7.4 | |
| y5077  without plant | 0 | 0.06±0.01 | 0.17±0.05 | | 0.02±0.01 | | 0.09±0.02 | |
| y5077  with plant | 0 | 5.4±0.64 | 7.7±0.45 | | 10.3±1.5 | | 14.5±1.26 | |

Supplementary table 2. Colonization of *M. hupehensis* roots surface (cfu g-1 fresh weight) by rhizobia. Phloridzin concentration applied in the experiment was 100μmol kg^-1^.

| Strains | – phloridzin | + phloridzin |
| --- | --- | --- |
| Control | 0 | 0 |
| y4071 | 2.16 × 10^5^ | 1.16 × 10^6^ |
| y5077 | 1.62 × 10^5^ | 2.82 × 10^7^ |
| y4009 | 2.97 × 10^5^ | 3.9 × 10^5^ |
| y4031 | 2.07 × 10^5^ | 6.75 × 10^5^ |

Supplementary table 3. Effects of rhizobia on root system architecture of *Malus hupehensis* under 200 μmol kg^-1^phloridzin induced stress.

|  | Control | phloridzin | rhizobia | phloridzin+rhizobia |
| --- | --- | --- | --- | --- |
| Length (cm) | 511±20.2a | 322±14.6b | 550±32.5a | 497±34a |
| Diameter (mm) | 0.48±0.02b | 0.33±0.02c | 0.57±0.014a | 0.45±0.03b |
| Volume (cm^3^) | 0.77±0.05a | 0.37±0.03b | 0.83±0.06a | 0.68±0.05a |
| Number of root tips | 1522±134a | 982±101c | 1332±74b | 1244±82b |
| Number of root forks | 3522±251a | 1291±147c | 3381±236b | 3192±127b |

Means with different letters within each row are significantly different (one-way

ANOVA, *p* < 0.05).

Supplementary table 4. Quantitative analysis of in situ H_2_O_2_ accumulation (relative DAB-stained area) in *Malus hupehensis* leaves under different treatments.

| Treatment | 5 Days (%) | 10 Days (%) | 15 Days (%) | 20 Days (%) | 30 Days (%) |
| --- | --- | --- | --- | --- | --- |
| Control | 4.2 ± 1.3 (I) | 5.1 ± 1.4 (I) | 6.8 ± 1.5 (I) | 8.5 ± 1.8 (I) | 18.4 ± 2.6 (II) |
| y5077 | 5.3 ± 1.1 (I) | 19.5 ± 2.8 (II) | 24.1 ± 3.3 (II) | 42.6 ± 4.2 (III) | 26.8 ± 3.9 (II) |
| Phloridzin | 6.5 ± 1.3 (I) | 28.3 ± 4.2 (II) | 76.5 ± 6.1 (IV) | 84.2 ± 5.2 (IV) | 95.6 ± 3.7 (V) |
| Phloridzin+y5077 | 4.8 ± 1.0 (I) | 8.7 ± 1.6 (I) | 22.4 ± 3.5 (II) | 27.9 ± 3.4 (II) | 48.5 ± 5.2 (III) |

Supplementary table 5: Physical and chemical characteristics of the soil.

| Soil properties |  |
| --- | --- |
| sand (%) | 49 |
| silt (%) | 26 |
| clay (%) | 25 |
| soil pH (1:1 soil/water ratio) | 7.2 |
| NO_3_-N (mg/kg) | 11.3 |
| NH_4_-N (mg/kg) | 14.7 |
| P (mg/kg) | 8.2 |
| organic mater content (%) | 1.13 |
| phloridzin | — |

‘—’ indicated the phloridzin was not been detected in the soil.

Supplementary table 6: Measurement on main parameters of phloridzin and melatonin by HPLC

|  | Retention time min | Linear equation | R2 | Detection limit mg L^-1^ | Linear range mg L^-1^ |
| --- | --- | --- | --- | --- | --- |
| phloridzin | 7.14 | Y=1.66×10^-6^X+0.214 | 0.9981 | 2.4 | 0-500 |
| melatonin | 3.53 | Y=4.23×10^-6^X+0.638 | 0.9994 | 1.7 | 0-1000 |

‘y’ indicated the concentration of phloridzin or melatonin mg L^-1^; ‘x’ indicated the peak area µAU*s.

Supplementary table 7: Sequences for primers used in qRT-PCR (Li et al. 2014; Wei et al. 2018)

| Sequences for primers used in qRT-PCR | |
| --- | --- |
| ascorbate-glutathione cycle | |
| *MdcAPX* | F: AACTACAAGGGATGAAGCC R: CAACGAGGATGATAACCAG |
| *MdDHAR1* | F: AGTGGACGGTTCCAGCAGA R: TTCCCATCCCGCAATCAC |
| *MdDHAR2* | F: CCACCATCAAACACCACCTT R: TTGGGAACAGTAACGGAAGC |
| *MdcGR* | F: GTTCAGCGACAAGGCGTAT R: TCAACCGATTTCCATTTCC |
| Melatonin synthesis |  |
| *MdTDC1* | F: TCACGCTGTGGTTGGAGGT R: CTGCATGCTCCTGAACCAAC |
| *MdT5H4* | F: TCGGTGACATGTTTGCTGC R: GGAAACCTTGGTCTGGCG |
| *MdAANAT2* | F: GAATCACCGTCCACGCTCC R: GAAATGCTTCCGATGTCCC |
| *MdASMT1* | F: AGAGGAGCGAGAAAGACTGGA R: CTAAAGAAAAACTTCAATGAGGGAT |
| *EF-1α* | F: ATTCAAGTATGCCTGGGTGC R: CAGTCAGCCTGTGATGTTCC |

**References:**

Li, C., Liang, B., Chang, C., Wei, Z., Zhou, S., Ma, F., 2016. Exogenous melatonin improved potassium content in Malus under different stress conditions. J Pineal Res. 61:218-29.

Wei, Z. Gao, T. Liang, B. Zhao, Q. Ma, F. Li, C., 2018. Effects of exogenous melatonin on methyl viologen-mediated oxidative stress in apple leaf. Int. J. Mol. Sci.19, 316

Supplementary Figure 1. Phloridzin degradation rate by the rhizobia isolates over 96 h in basal mineral medium.

Supplementary Figure 2. Photographs showing the effects of rhizobia on the root architecture of *Malus hupehensis* under 200 μmol·kg⁻¹ phlorizin stress.

Supplementary Figure 3. Effect of rhizobia inoculation on expression patterns of ascorbate-glutathione cycle genes in *M.hupehensis* under phloridzin stress. A, expression of *MdcAPX*; B, expression of *MdDHAR1*; C, expression of *MdDHAR2*; D, expression of *MdcGR*. Data are the means (SD) of three replicates. Different lowercase letters above the bars indicate statistically significant differences between treatments according to Tukey’s HSD test (*p* < 0.05).

Supplementary Figure 4. Comparison of rhizosphere bacterial and fungal alpha diversity indices under different treatments. Alpha diversity indices include (A) Bacterial Chao1 index, (B) Bacterial Shannon index, (C) Fungal Chao1 index, and (D) Fungal Shannon index.Treatments: Data are presented as the mean±standard deviation (SD) (n = 5). Statistical significance between groups was determined by the independent t-test. Asterisks indicate significant differences (**p* < 0.05; ** *p* < 0.01).

Supplementary Figure 5. Spearman's rank correlation matrix linking key rhizosphere microbial taxa to host physiological and environmental parameters. The X-axis represents soil chemical and plant physiological properties, and the Y-axis represents the relative abundances of core bacterial and fungal phyla. The color scale denotes Spearman's correlation coefficient (ρ). Asterisks indicate statistical significance: * *P* < 0.05, ** *P* < 0.01. Note: Spearman’s rank correlations were calculated using individual pot-level data (n = 5 independent biological replicates per treatment group) rather than treatment means.
